# Mechanisms of fetal growth restriction in gestational cholestasis: role of gut microbiota and placental redox

**DOI:** 10.3389/fvets.2026.1815498

**Published:** 2026-03-20

**Authors:** Zhenhua Xue, Qi Han, Huigang Han, Laiqing Yan, Xiao Ma, Pengyun Ji, Bingyuan Wang, Lu Zhang, Likai Wang, Guoshi Liu

**Affiliations:** 1 State Key Laboratory of Animal Biotech Breeding, Frontiers Science Center for Molecular Design Breeding, College of Animal Science and Technology, China Agricultural University, Beijing, China; 2 Department of Hepatobiliary Surgery and Liver Transplant Center, The First Hospital of Shanxi Medical University, Taiyuan, China; 3 College of Animal Science, Xinjiang Agricultural University, Xinjiang, China

**Keywords:** Cholestasis, IUGR, metabolome, microbiota, oxidative stress

## Abstract

**Introduction:**

Intrahepatic cholestasis of pregnancy (ICP) is a cholestatic liver disorder associated with substantial fetal morbidity, including preterm birth, fetal distress, and even intrauterine demise. Although prior studies have documented structural and transcriptional alterations in the placenta during ICP, the mechanistic underpinnings linking maternal cholestasis to adverse fetal outcomes remain incompletely elucidated.

**Methods:**

In this study, a murine model of ICP was established by feeding pregnant C57BL/6 mice a 0.1% DDC (3,5-dicarboxylic acid-1,4-dihydrocollidine) diet from E0.5 to E18.5. We assessed fetal growth and employed multi-omics approaches, including placental transcriptome sequencing, maternal gut microbiome profiling, and serum/placental metabolome analysis.

**Results:**

Placental transcriptome sequencing revealed that ICP significantly downregulated the expression of antioxidant-related genes including *Mgst1, Gstt1, Ggt1, Gpx8, Gstk1*, and *GSTA4* leading to reduced total antioxidant capacity in placental tissue and elevated levels of malondialdehyde (MDA), a marker of lipid peroxidation. Furthermore, ICP disrupted the maternal gut microbiota, resulting in decreased production of antioxidant microbial metabolites such as valeric acid and erythritol. This deficiency further aggravated oxidative damage in the placenta.

**Discussion:**

Collectively, our findings uncover a novel gut microbiota-placenta axis driven by cholestasis, which contributes to fetal IUGR. The maternal cholestasis induces gut dysbiosis, which diminishes the production of valeric acid and erythritol. The deficiency of these metabolites, coupled with a direct suppression of the placental Nrf2/Keap1 antioxidant signaling pathway by cholestasis, leads to placental oxidative stress. This oxidative damage impairs placental function, ultimately resulting in fetal growth restriction. Disrupting this pathogenic cycle may offer a promising therapeutic strategy for preventing or treating ICP-related reproductive disorders.

## 1 Introduction

Cholestasis represents a core pathological feature of several hepatobiliary disorders, including primary biliary cholangitis (PBC) and primary sclerosing cholangitis (PSC) (1). It is defined by impaired bile formation or flow, resulting in the accumulation of bile acids and other cytotoxic compounds in the liver and systemic circulation (2). This disruption typically stems from injury to hepatocytes or cholangiocytes, compromising bile acid synthesis, transport, or excretion, and thereby contributing to progressive hepatobiliary dysfunction in both acute and chronic settings (3). Sustained cholestasis triggers oxidative stress, inflammation, and activation of fibrogenic pathways, which can culminate in liver fibrosis, cirrhosis, and an elevated risk of hepatocellular or cholangiocellular carcinoma (4).

Intrahepatic cholestasis of pregnancy (ICP) is the most common liver-specific disorder of gestation, typically emerging in the second or third trimester. Clinically, it presents with intense pruritus and biochemical evidence of cholestasis, notably elevated serum total bile acids (TBA) and alanine aminotransferase (ALT) (5). During normal pregnancy, hepatic adaptations support fetal metabolic demands, including a physiological rise in circulating bile acids. However, in ICP, this balance is disrupted, leading to pathologically high bile acid levels that pose significant risks (6). Maternal ICP-particularly when TBA ≥40 μmol/L, is strongly associated with adverse outcomes such as spontaneous preterm delivery, fetal distress, and intrauterine fetal demise (7). Alarmingly, emerging evidence suggests that prenatal exposure to cholestasis may exert long-term programming effects: offspring exhibit increased susceptibility to metabolic syndrome, obesity, type 2 diabetes, and liver fibrosis in adulthood (8, 9). While maternal bile duct ligation (BDL) and exposure to environmental toxins like di-(2-ethylhexyl) phthalate have been used to model ICP and replicate fetal growth restriction, the utility of dietary 0.1% DDC (3,5-diethoxycarbonyl-1,4-dihydrocollidine)-a well-established cholestatic agent, remains underexplored in the context of pregnancy (9, 10).

Intrauterine growth restriction (IUGR) is defined as the failure of a fetus to reach its genetically determined growth potential. It poses significant short-term risks to neonatal health and is strongly linked to long-term adverse outcomes in adulthood, including heightened susceptibility to metabolic syndrome, cardiovascular disease, and type 2 diabetes mellitus (11). Despite growing recognition of the fetal risks associated with intrahepatic cholestasis of pregnancy (ICP), the precise contribution of gestational cholestasis to IUGR remains incompletely understood. The placenta, a transient but vital organ-functions as the central interface for maternal–fetal exchange, mediating nutrient delivery, gas transfer, waste elimination, and endocrine signaling throughout gestation (12). Emerging evidence suggests that cholestasis may compromise uteroplacental perfusion, induce acute fetal hypoxia, and disrupt placental barrier integrity, thereby contributing to adverse pregnancy outcomes (13). Notably, cholestatic placentas exhibit marked downregulation of key bile acid transporters, including organic anion transporting polypeptide 1A2 (OATP1A2) and OATP1B3, potentially impairing bile acid clearance and exacerbating fetal exposure to toxic metabolites (14). However, the molecular and functional mechanisms by which maternal cholestasis impairs placental physiology remain elusive.

To address this gap, we established a murine model of gestational cholestasis and systematically evaluated its effects on placental function and fetal growth. Integrating multi-omics approaches including placental transcriptome sequencing, maternal gut microbiome profiling, and serum and placental metabolome analysis we aimed to delineate the pathophysiological pathways linking cholestasis to IUGR. Our findings reveal that cholestasis induces placental oxidative stress, disrupts antioxidant defenses, and alters microbial-derived metabolite profiles, collectively impairing fetal development. These insights not only advance our understanding of the mechanistic basis of cholestasis-related reproductive dysfunction but also offer potential biomarkers and therapeutic targets for early intervention in high-risk pregnancies.

## 2 Materials and methods

### 2.1 Animals and experimental design

All animal procedures were approved by the Animal Welfare Committee of China Agricultural University (Approval No. AW30604202-1-1) and conducted in accordance with institutional guidelines. Twelve 8-week-old female C57BL/6 mice were housed under specific pathogen-free conditions with a 12-h light/dark cycle, ambient temperature of 21 ± 2 °C, and relative humidity of 45 ± 5%. Mice were mated with fertile males, and the presence of a vaginal plug was designated as embryonic day 0.5 (E0.5). Pregnant females were then randomly assigned to two groups (*n* = 6 per group): the control group received a standard chow diet, while the cholestasis group was fed a diet supplemented with 0.1% 3,5-diethoxycarbonyl-1,4-dihydrocollidine (DDC) from E0.5 until E18.5. At E18.5, mice were anesthetized with sodium pentobarbital, and maternal liver, placental, and fetal tissues were collected, weighed, and processed for downstream analyses. Maternal serum and cecal contents were also harvested. Reproductive outcomes-including fetal weight, placental weight, and placental efficiency (fetal weight/placental weight)-were recorded for all conceptuses.

### 2.2 Histopathological analysis

Liver tissues were fixed in 4% paraformaldehyde, dehydrated through a graded ethanol series, embedded in paraffin, and sectioned at 5 μm. Sections were dewaxed, rehydrated, stained with hematoxylin and eosin (H&E), dehydrated, mounted, and examined under a light microscope.

### 2.3 Placental RNA sequencing (RNA-Seq)

Placental tissues from fetuses with weights closest to the litter mean were selected: four from the control group and six from the DDC group. Total RNA was extracted using TRIzol reagent (Ambion, #15596026). RNA integrity (RIN ≥6.5), purity (OD260/280 = 1.8–2.2; OD260/230 ≥2.0), and rRNA ratio (28S:18S ≥1.0) were verified using an Agilent 2100 Bioanalyzer and NanoDrop spectrophotometer. Libraries were prepared with the TruSeq™ RNA Sample Prep Kit (Illumina). Briefly, mRNA was reverse-transcribed into double-stranded cDNA (SuperScript kit, Invitrogen), fragmented, adapter-ligated, and PCR-amplified. Fragments of 200–300 bp were size-selected using DNA Clean Beads. Libraries were quantified with PicoGreen and sequenced on an Illumina HiSeq X Ten/NovaSeq 6000 platform (PE150). Differentially expressed genes (DEGs) were identified using DESeq2 with thresholds of |log_2_ (Fold Change)| ≥0.585 (equivalent to Fold Change ≥1.5) and *p* < 0.05.

### 2.4 Serum untargeted metabolomics

Maternal serum samples (*n* = 6 per group) were analyzed via liquid chromatography–mass spectrometry (LC-MS). Raw data underwent preprocessing (missing value imputation, normalization), followed by multivariate analysis: principal component analysis (PCA) for global trends and orthogonal partial least squares discriminant analysis (OPLS-DA) for group separation. Metabolites were annotated using HMDB and METLIN databases. Differential metabolites were defined by VIP >1.0 (from OPLS-DA) and *p* < 0.05 (Student's *t*-test). Pathway enrichment was performed using the KEGG database.

### 2.5 Quantitative real-time PCR (qRT-PCR)

Total RNA from placental tissues was reverse-transcribed using PrimeScript™ RT Master Mix (TaKaRa). qPCR was performed with SYBR Green Master Mix (Roche) on a real-time system. Gene expression was normalized to Gapdh and calculated via the 2^−Δ*ΔCT*^ method. The sequences of primers are provided in Table S1.

### 2.6 Biochemical assays

Placental tissues were homogenized (1:9 w/v) in ice-cold saline, centrifuged (12,000 rpm, 5 min), and supernatants used to assess total antioxidant capacity (T-AOC) with a commercial kit (Nanjing Jiancheng, A015-3-1). For malondialdehyde (MDA) measurement, homogenates were centrifuged at 10,000–12,000 × g for 10 min, and MDA levels were quantified using a Beyotime assay kit (S0131S).

### 2.7 16S rDNA amplicon sequencing

Cecal contents from four dams per group were used for microbiome analysis. Genomic DNA was extracted (QIAGEN kit) and quantified (Qubit 2.0). The V3–V4 hypervariable regions of the 16S rDNA gene were amplified with barcoded primers, purified (GeneJET Gel Extraction Kit), and used to construct libraries (NEBNext Ultra DNA Library Prep Kit). Sequencing was performed on an Illumina platform (250 bp paired-end). Raw reads were quality-filtered, merged, and denoised to generate amplicon sequence variants (ASVs). Alpha diversity (Simpson index), beta diversity (Bray–Curtis PCoA), and Microbial Dysbiosis Index (MDI) were calculated. Differentially abundant taxa were identified by LEfSe (LDA >2.5, *p* < 0.05).

### 2.8 Statistical analysis

Data are presented as Mean ± SEM. Normality and homogeneity of variance were confirmed. Comparisons between two groups were performed using two-tailed Student's t-test (GraphPad Prism 7.0). *p* < 0.05 was considered statistically significant.

## 3 Results

### 3.1 Cholestasis and hepatic injury in pregnant mice were induced by DDC supplementation

**Figure 1:**
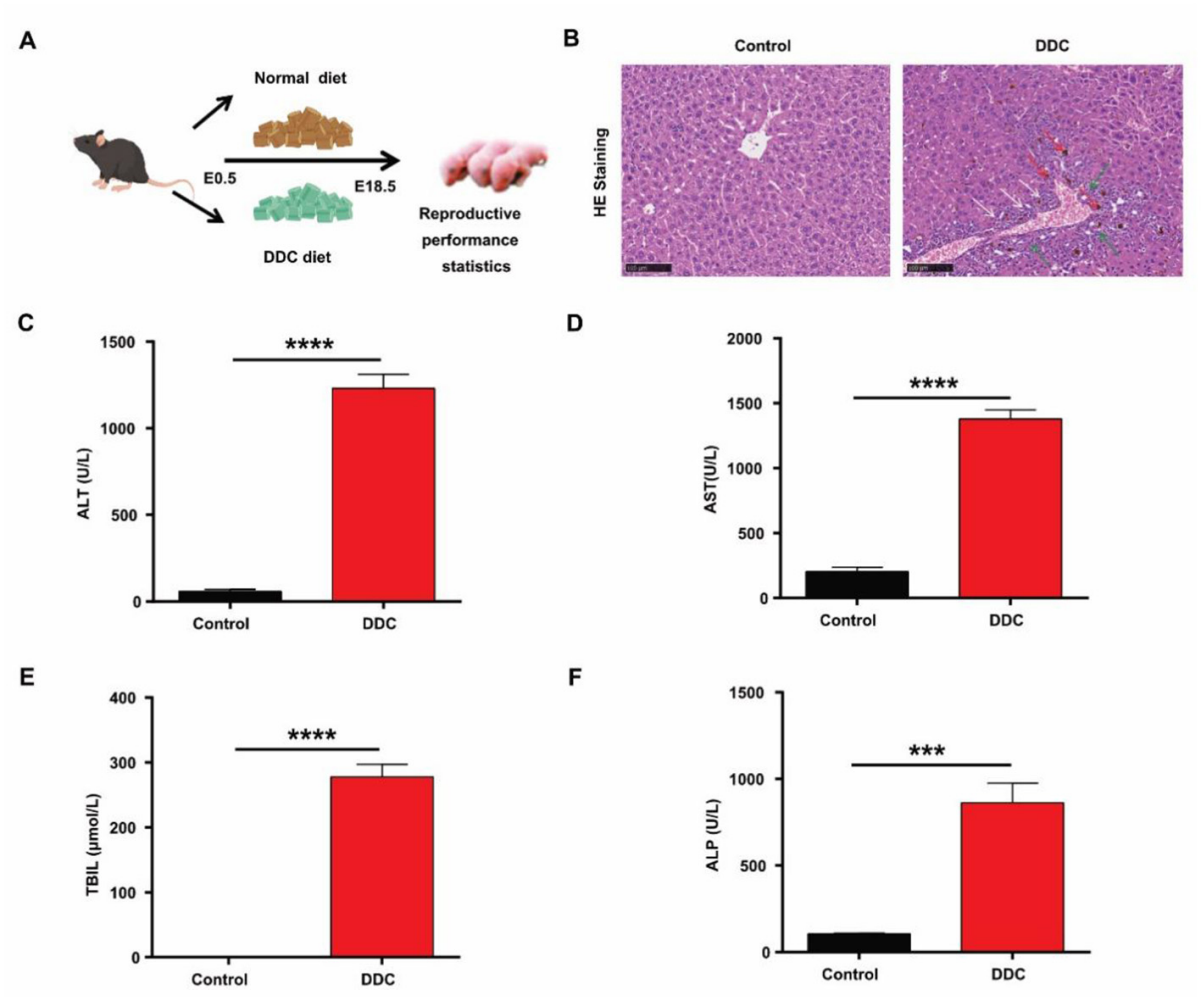
Effects of DDC diet on maternal cholestasis and liver function. **(A)** Experimental design for treating mice with DDC. **(B)** HE staining of liver tissue. Scale bars, 100 μm. White arrows indicate bile duct hyperplasia, red arrows indicate porphyrin, and green arrows indicate inflammatory cells. **(C)** Serum level of ALT. **(D)** Serum level of AST. **(E)** Serum level of TBIL. **(F)** Serum level of ALP. Data are presented as means ± SEM. *n* = 6. ***p < 0.001, ****p < 0.0001.

To model intrahepatic cholestasis of pregnancy (ICP), pregnant C57BL/6 mice were fed a diet containing 0.1% DDC (3,5-dicarboxylic acid-1,4-dihydrocollidine) from embryonic day 0.5 (E0.5) to E18.5 (Figure 1A). Histopathological analysis of maternal liver tissue revealed hallmark features of cholestasis: porphyrin plug–induced bile duct obstruction, marked bile duct hyperplasia, periductal inflammatory infiltration, and early fibrotic changes (Figure 1B). Serum biochemical analysis confirmed significant hepatobiliary dysfunction in the DDC-treated group. As shown in Figures 1C, D, levels of alanine aminotransferase (ALT, 204.3 ± 34.07 vs. 1,379 ± 70.4 U/L, *p* < 0.0001) and aspartate aminotransferase (AST, 58.25 ± 11.5 *vs*. 1,230 ± 81.27 U/L, *p* < 0.0001)-markers of hepatocellular damage-were markedly elevated. Additionally, serum concentrations of total bilirubin (TBIL; 0.95 ± 0.06 *vs*. 277.9 ± 19.35 μmol/L, *p* < 0.0001) and alkaline phosphatase (ALP, 106.0 ± 3.42 *vs*. 861.3 ± 114.3 U/L, *p* < 0.0001) -canonical indicators of cholestasis, were significantly increased compared to controls (Figures 1E, F). Together, these histological and serological results demonstrate that dietary DDC administration effectively induces a robust and reproducible model of gestational cholestasis accompanied by hepatic injury in pregnant mice.

### 3.2 Maternal cholestasis causes fetal intrauterine growth restriction (IUGR)

**Figure 2:**
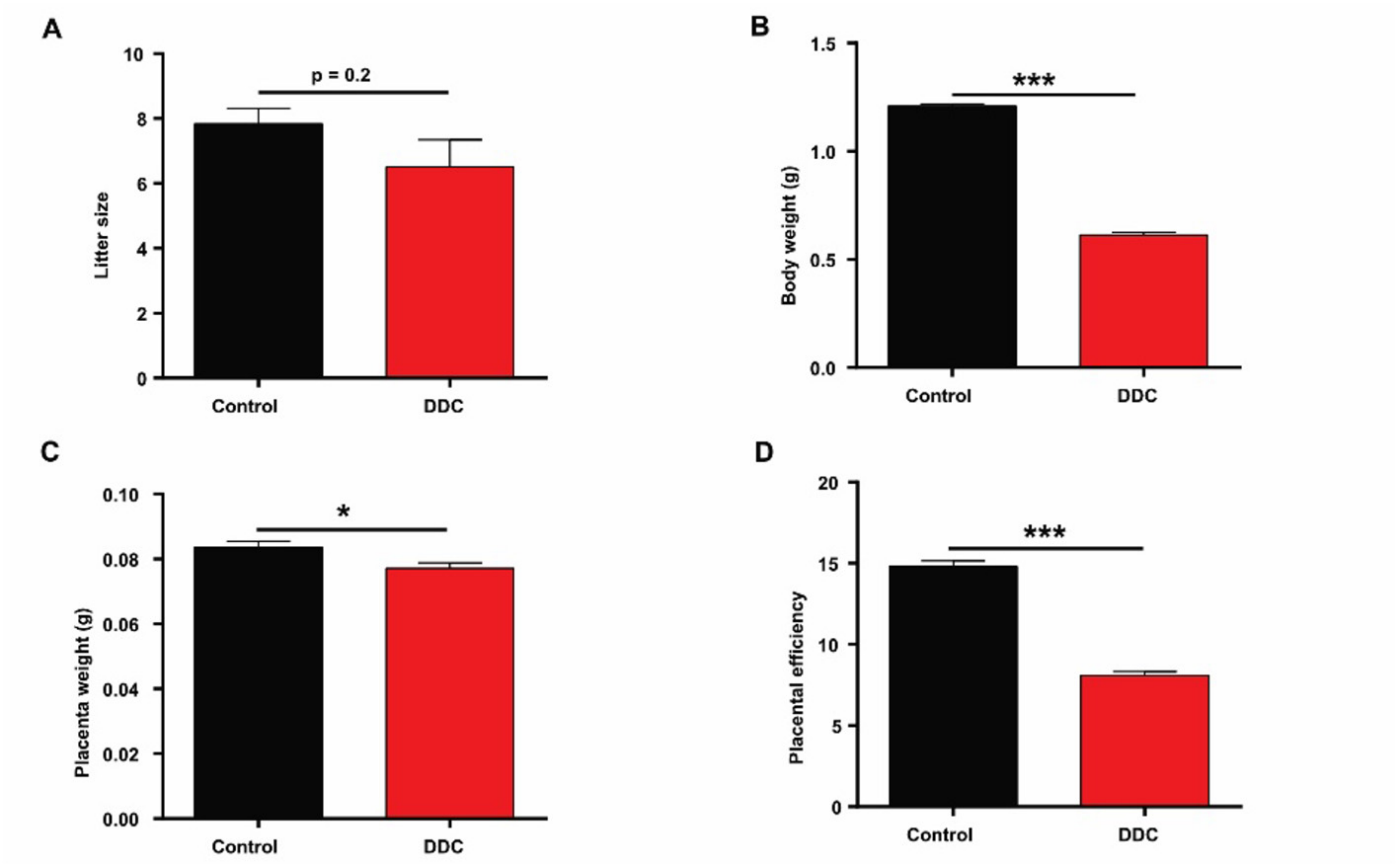
Statistics of reproductive performance data of mice. **(A)** Average litter size (*n* = 6). **(B)** Average body weight (*n* = 47 for Control, *n* = 39 for DDC group). **(C)** Average placenta weight (*n* = 47 for Control, *n* = 39 for DDC group). **(D)** Average placenta efficiency (placenta efficiency = body weight/placenta weight, *n* = 47 for Control, *n* = 39 for DDC group). Data are presented as means ± SEM. *p < 0.05. ***p < 0.001.

To evaluate the impact of gestational cholestasis on fetal development, reproductive outcomes were assessed at embryonic day 18.5 (E18.5). While maternal DDC exposure did not significantly alter litter size (7.83 ± 0.48 *vs*. 6.5 ± 0.85, *p* > 0.05; Figure 2A), it profoundly impaired fetal growth. Fetuses from DDC-treated dams exhibited significantly lower body weights compared to controls (Figure 2B; 1.21 ± 0.01 *vs*. 0.61 ± 0.01 g, *p* < 0.001). Concurrently, placental weight was reduced in the DDC group (0.08 ± 0.001 *vs*. 0.07 ± 0.001, *p* < 0.05; Figure 2C), and placental efficiency-defined as the ratio of fetal weight to placental weigh, which was markedly diminished (14.81 ± 0.36 *vs*. 8.09 ± 0.24 g, *p* < 0.001; Figure 2D). This reduction in placental efficiency indicates compromised nutrient transfer capacity and impaired placental function. Collectively, these findings demonstrate that maternal cholestasis induced by DDC administration leads to intrauterine growth restriction (IUGR) without affecting the number of conceptuses, highlighting the placenta as a critical target of cholestatic injury during pregnancy.

### 3.3 Maternal cholestasis suppresses placental antioxidant defenses and induces oxidative stress

**Figure 3:**
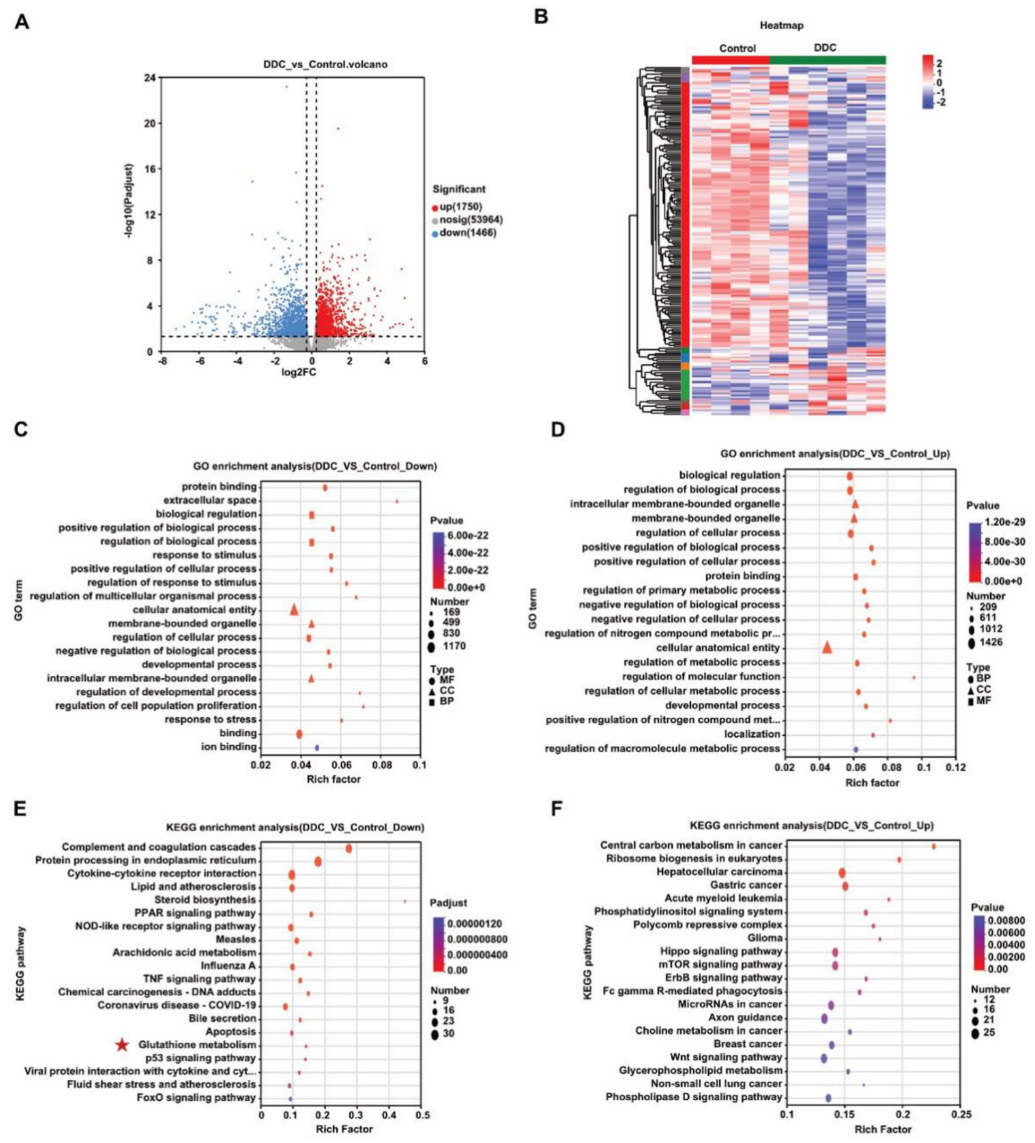
Placental transcriptome sequencing analysis. **(A)** Volcano plots of differential genes between DDC and Control groups. **(B)** Differential gene clustering heat map. **(C)** GO enrichment analysis of differentially upregulated genes. **(D)** GO enrichment analysis of differentially downregulated genes. **(E)** KEGG enrichment analysis of differentially upregulated genes. **(F)** KEGG enrichment analysis of differentially downregulated genes.

**Figure 4:**
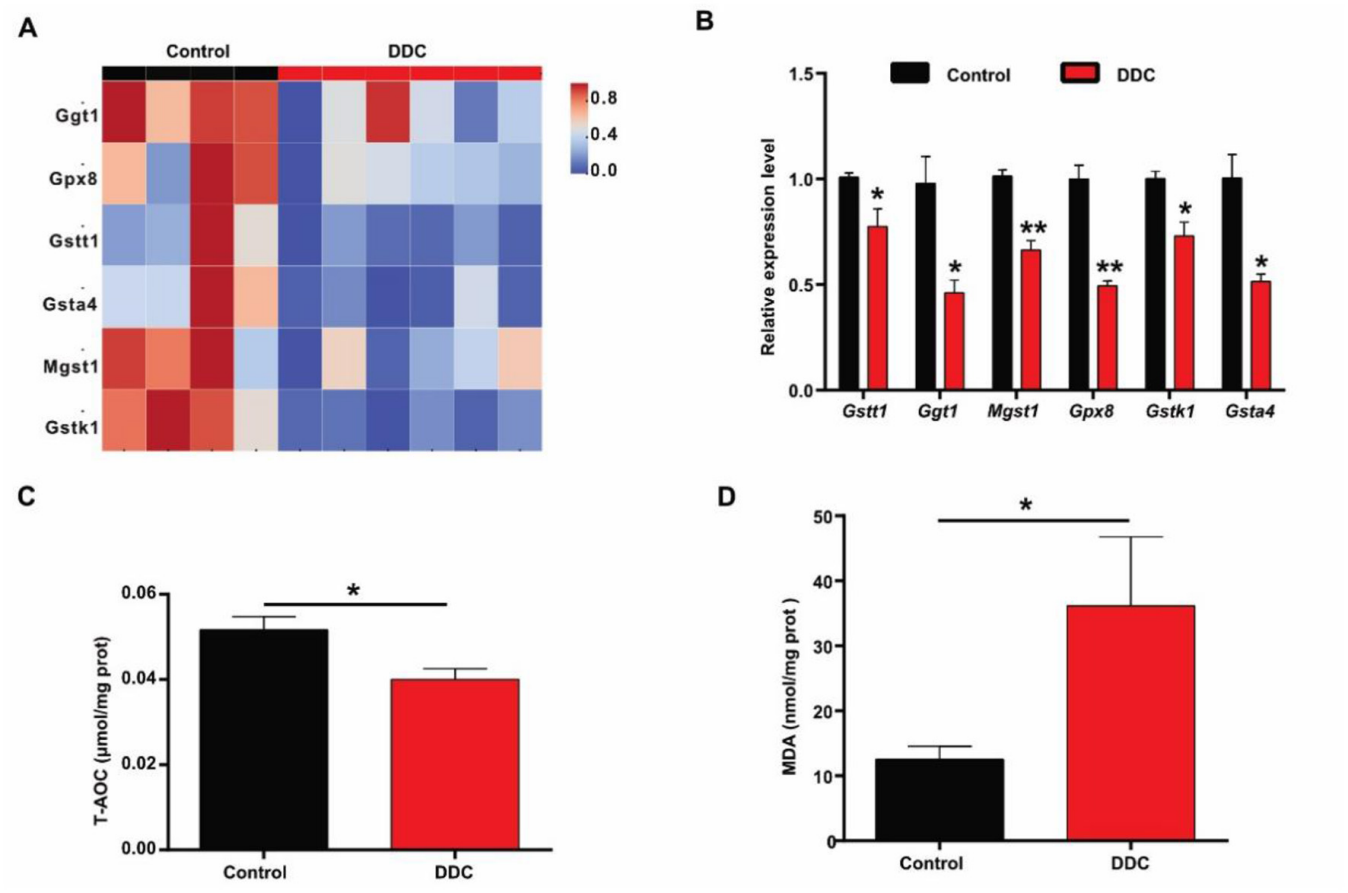
Detection of the antioxidant capacity of the placenta. **(A)** Differential genes related to antioxidation. **(B)** The mRNA levels of genes associated with antioxidant (*n* = 6). **(C)** The total antioxidant capacity of placental tissue (*n* = 6). **(D)** MDA level in placental tissue (*n* = 6). Data are presented as means ± SEM. *p < 0.05.

To investigate the molecular basis of placental dysfunction in gestational cholestasis, we performed transcriptome RNA sequencing on placental tissues at E18.5. Comparative analysis between control and DDC-exposed groups revealed 3,216 differentially expressed genes (DEGs), with 1,466 downregulated and 1,750 upregulated (Figures 3A, B). Gene Ontology (GO) and KEGG pathway enrichment analyses showed that downregulated genes were significantly associated with extracellular space organization and, notably, key metabolic pathways including glutathione metabolism, PPAR signaling, and steroid biosynthesis (Figures 3C–F). Within the glutathione metabolism pathway, multiple antioxidant genes-including *Mgst1, Gstt1, Ggt1, Gpx8, Gstk1*, and *GSTA4*-were consistently downregulated in the DDC group (Figure 4A), suggesting a compromised antioxidant defense system. We next validated these transcriptional changes by qRT-PCR, which confirmed significantly reduced mRNA levels of all six genes (*p* < 0.05; Figure 4B). Critically, this transcriptional suppression translated into measurable functional deficits: the total antioxidant capacity (T-AOC) of placenta was significantly diminished (0.05 ± 0.003 *vs*. 0.04 ± 0.002, *p* < 0.05; Figure 4C), while levels of malondialdehyde (MDA), which is a marker of lipid peroxidation and oxidative damage—were markedly elevated (12.54 ± 2.02 *vs*. 36.16 ± 10.60, *p* < 0.05; Figure 4D). These findings demonstrate that maternal cholestasis disrupts placental redox homeostasis by suppressing critical antioxidant pathways at both the transcriptional and functional levels, culminating in oxidative stress that likely underlies placental insufficiency and fetal intrauterine growth restriction (IUGR).

### 3.4 Maternal gut microbiota is disrupted by gestational cholestasis

**Figure 5:**
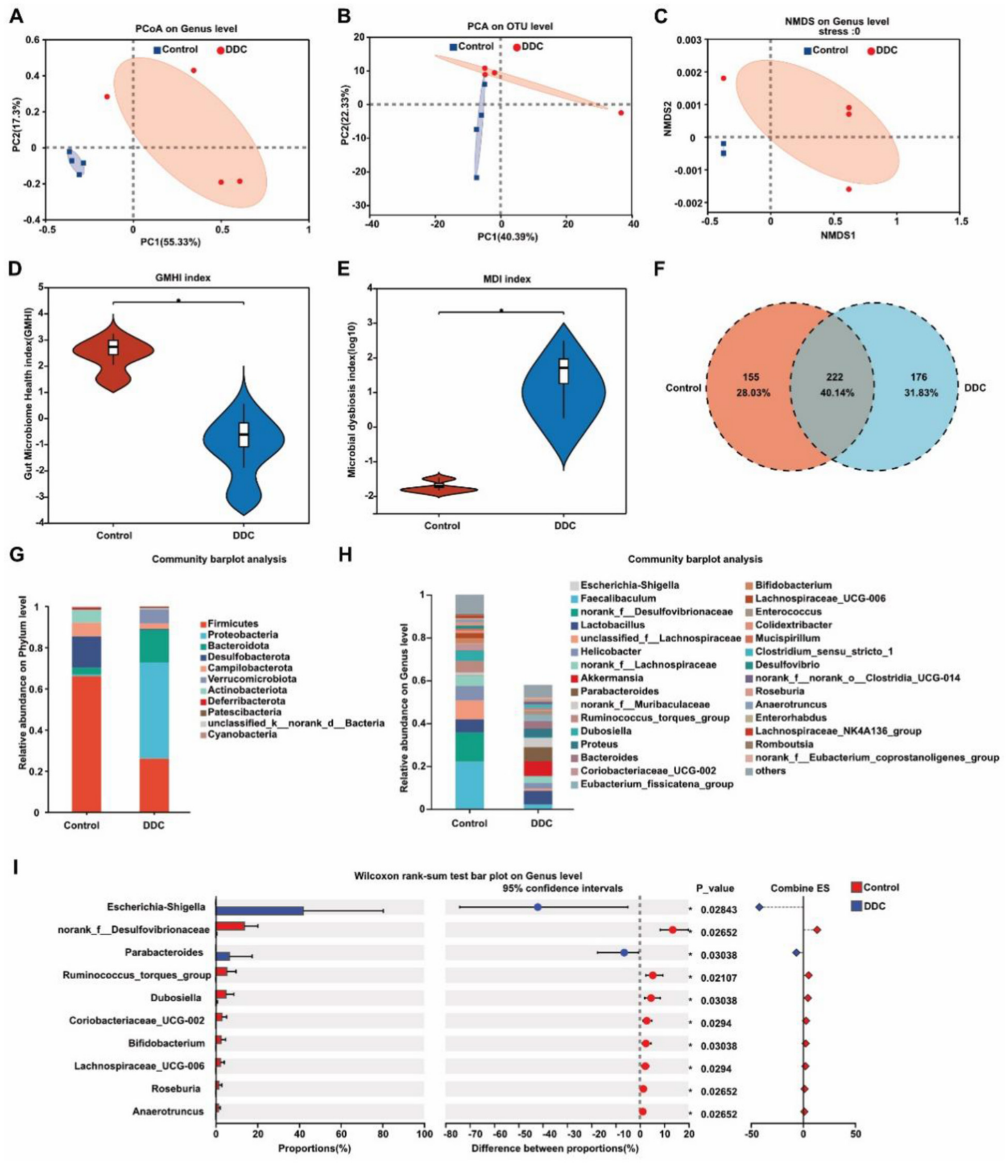
Analysis results of the maternal intestinal microbiota of pregnant mice in the control group and the DDC group **(A)** Principal component analysis. **(B)** Principal coordinate analysis. **(C)** Non-metric multidimensional scaling analysis. **(D)** Gut microbial health index. **(E)** Gut microbial dysbiosis index. **(F)** Species Venn diagram analysis. **(G, H)** Analysis of microbial composition and abundance. **(I)** Wilcoxon rank-sum test bar plot on genus level.

To investigate the impact of cholestasis on the maternal gut ecosystem during pregnancy, we performed 16S rDNA amplicon sequencing on cecal contents from control and DDC-exposed dams at E18.5. Beta-diversity analyses-including principal coordinate analysis (PCoA), principal component analysis (PCA), and non-metric multidimensional scaling (NMDS)-revealed significant separation in microbial community structure between the two groups (Figures 5A–C), indicating that gestational cholestasis profoundly alters gut microbiota composition. Quantitative assessment of microbial health showed a marked reduction in the gut microbial health index and a concurrent increase in the microbial dysbiosis index in the DDC group (Figures 5D, E). While 222 bacterial taxa were shared between groups, 155 operational taxonomic units (OTUs) were unique to controls and 176 to the DDC group (Figure 5F), underscoring substantial community divergence. At the genus level, cholestasis induced specific shifts in microbial abundance. The DDC group exhibited significant enrichment of *g_Escherichia-Shigella* and *g_Parabacteroides*, both of which have been associated with inflammation and metabolic dysfunction. Conversely, beneficial or homeostasis-promoting genera-including *g_norank_f_Desulfovibrionaceae* and *g_unclassified_f_Lachnospiraceae*-were markedly depleted (Figures 5G, H). These results demonstrate that maternal cholestasis during pregnancy drives gut dysbiosis characterized by loss of commensal taxa and expansion of potentially pathobiontic genera, thereby disrupting the intestinal microbial balance essential for maintaining metabolic and immune homeostasis in gestation.

### 3.5 Gestational cholestasis reprograms maternal metabolism and depletes circulating antioxidant metabolites

**Figure 6:**
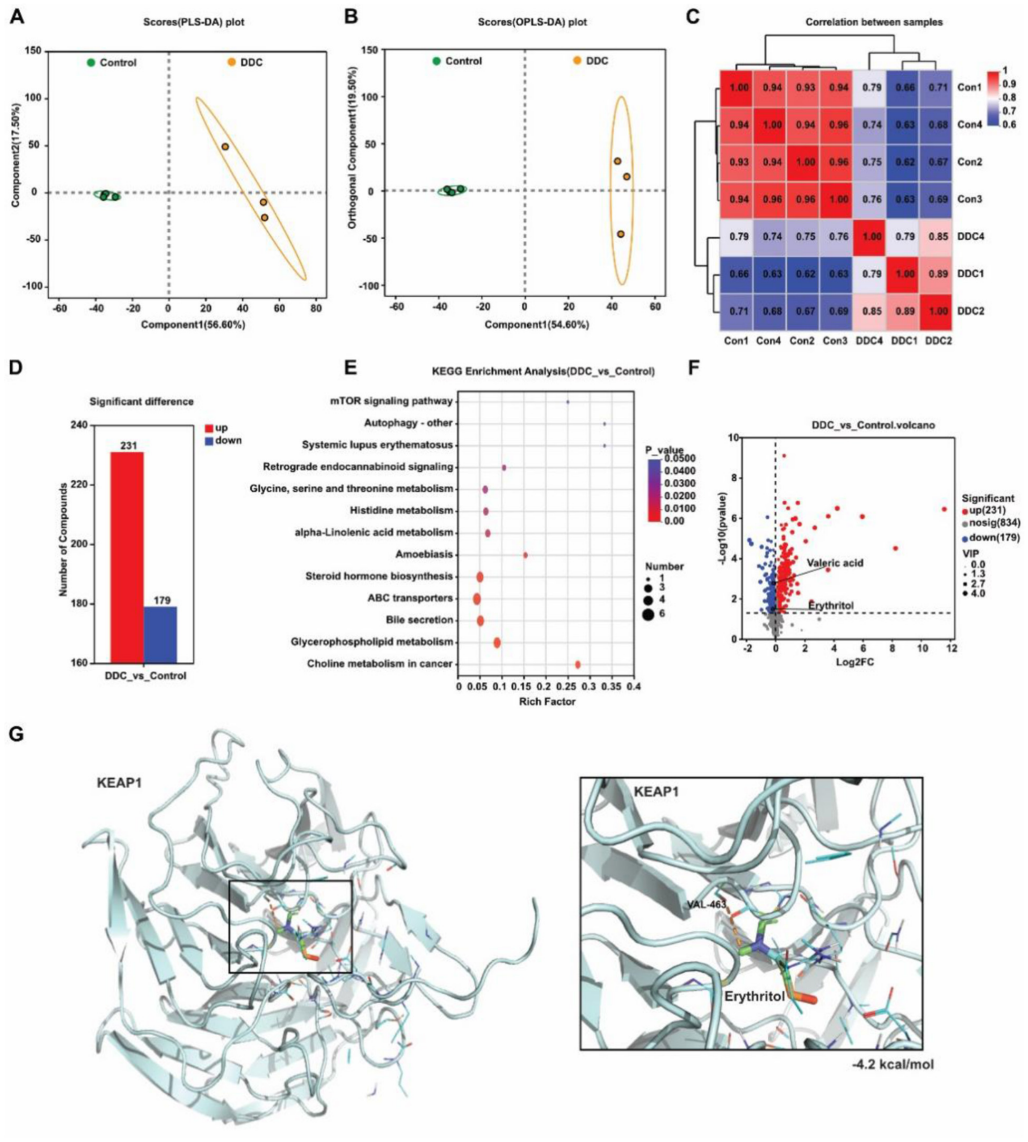
Analysis of maternal serum non-targeted metabolomics sequencing results. **(A)** Principal component analysis. **(B)** orthogonal partial least squares discriminant analysis. **(C)** Sample correlation heat map. **(D)** Statistics of differential metabolites between the control group and the DDC group. **(E)** KEGG enrichment analysis of differential metabolites. **(F)** Volcanic map of target metabolites. **(G)** Molecular docking of erythritol on KEAP1.

To characterize the systemic metabolic consequences of gestational cholestasis, we performed untargeted metabolomic profiling of maternal serum at E18.5. Quality control metrics confirmed high reproducibility within groups, while principal component analysis (PCA) and orthogonal partial least squares discriminant analysis (OPLS-DA) revealed clear separation between control and DDC-treated mice, indicating profound metabolic reprogramming (Figures 6A–C). A total of 410 differentially abundant metabolites were identified (*p* < 0.05, VIP >1.0), including 231 upregulated and 179 downregulated compounds (Figure 6D). KEGG pathway enrichment analysis highlighted significant perturbations in bile secretion, ABC transporters, and steroid hormone biosynthesis (Figure 6E)-pathways directly linked to hepatic function and xenobiotic clearance. Notably, levels of two microbially derived metabolites with established antioxidant properties were significantly reduced in the DDC group: valeric acid, a short-chain fatty acid produced by commensal gut bacteria through dietary fiber fermentation, and erythritol, a sugar alcohol implicated in cellular stress resistance (Figure 6F). Given the known role of erythritol in modulating redox signaling, we further explored its potential interaction with the Nrf2–Keap1 pathway-a master regulator of antioxidant responses. Molecular docking simulations indicated that erythritol can bind to the Kelch domain of Keap1 with high affinity, potentially competing with Nrf2 and thereby promoting Nrf2 release and activation (Figure 6G). These findings suggest that gestational cholestasis not only disrupts core metabolic pathways but also depletes circulating antioxidant metabolites, which may impair systemic and placental defense mechanisms against oxidative stress—thereby contributing to fetal growth restriction.

### 3.6 Gut microbiota-metabolite axis links dysbiosis to antioxidant deficiency in gestational cholestasis

**Figure 7:**
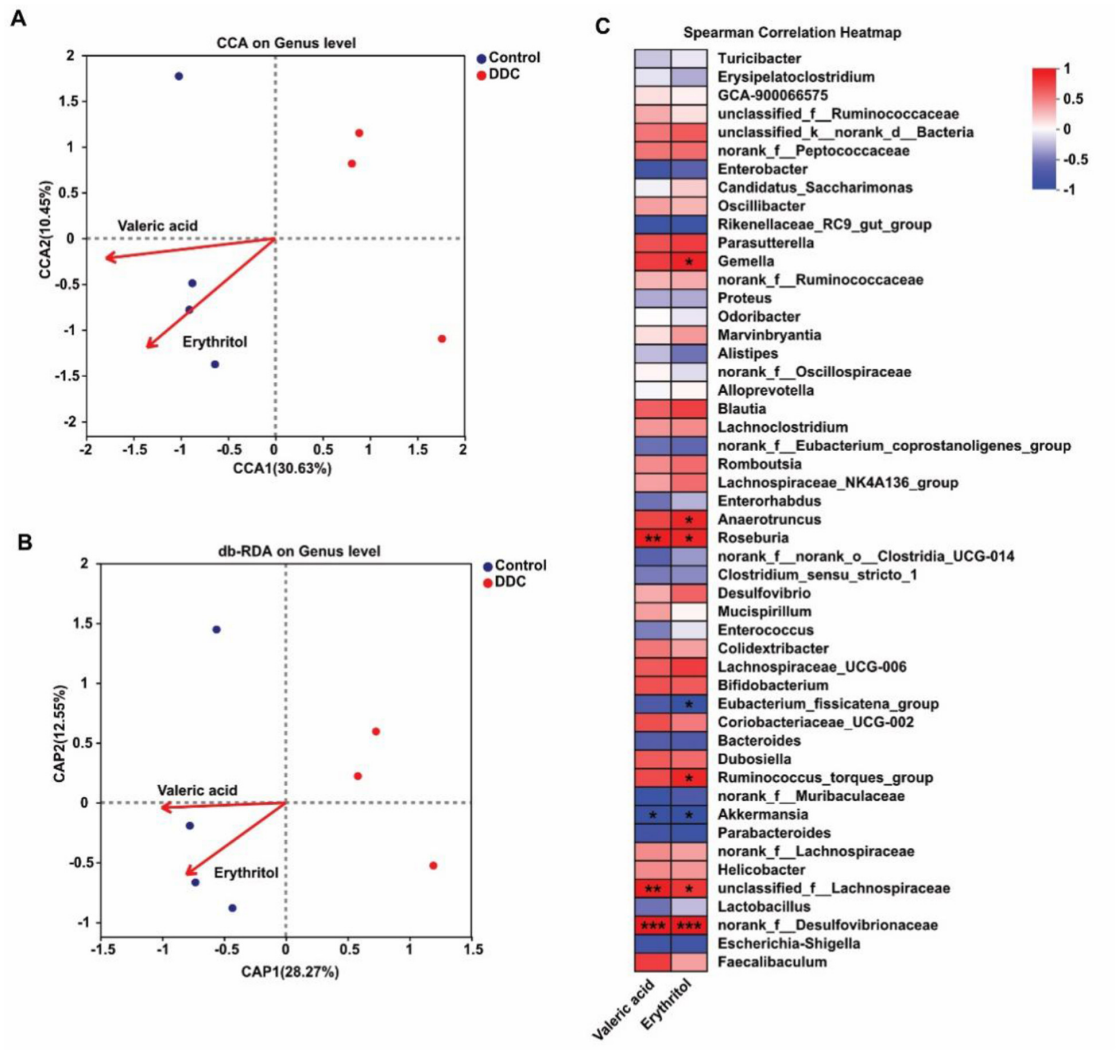
Correlation analysis of maternal intestinal microbiota and target metabolites. **(A)** CCA shows the relationship between the gut microbiota and metabolites at the genus level. **(B)** db-RDA analyzes the relationship between the gut microbiota and metabolites at the genus level. **(C)** Spearman correlation analysis of differential intestinal microbiota and metabolites.

To explore the functional interplay between cholestasis-induced gut dysbiosis and systemic metabolic alterations, we examined associations between differentially abundant gut microbial taxa and key antioxidant metabolites-specifically valeric acid and erythritol-which were significantly depleted in the DDC group. Canonical correspondence analysis (CCA) and distance-based redundancy analysis (db-RDA) revealed that microbial community structure at the genus level strongly correlated with metabolite profiles, particularly in the control group (Figures 7A, B), suggesting a tight host–microbe–metabolite relationship under healthy conditions that is disrupted during cholestasis. Spearman's correlation analysis further identified specific microbial–metabolite relationships: both valeric acid and erythritol levels showed significant positive correlations with the relative abundances of *g__norank_f__Desulfovibrionaceae* and *g__unclassified_f__Lachnospiraceae*-taxa that were markedly reduced in cholestatic mice. In contrast, *g__Akkermansia*, which was elevated in the DDC group, exhibited a significant negative correlation with both metabolites (Figure 7C). These findings provide compelling evidence that gestational cholestasis disrupts the symbiotic relationship between the maternal gut microbiota and host metabolism, leading to diminished production of microbially derived antioxidant metabolites. This microbiota-metabolite axis may represent a critical mechanistic link between intestinal dysbiosis and systemic oxidative stress in pregnancy-related cholestasis.

## 4 Discussion

Intrahepatic cholestasis of pregnancy (ICP) is a liver disorder of gestation characterized by maternal pruritus, elevated serum bile acids, and abnormal liver enzymes, and it is strongly associated with adverse perinatal outcomes, including preterm birth, fetal distress, and intrauterine growth restriction (IUGR) (15). The placenta-critical for nutrient exchange, endocrine signaling, and immune modulation-is particularly vulnerable to cholestatic injury (16–18). Histopathological studies in ICP patients have revealed placental abnormalities such as chorionic vessel constriction, reduced intervillous space, and increased syncytial knotting (19–21), all of which compromise fetal perfusion and development. Consistent with prior experimental models using estrogen-induced cholestasis (22), our DDC-fed pregnant mice exhibited significant reductions in fetal weight and placental efficiency, confirming that maternal cholestasis directly impairs fetal growth without necessarily altering litter size.

A growing body of evidence implicates oxidative stress as a central mechanism linking ICP to placental dysfunction (23). Under physiological conditions, the placenta maintains redox homeostasis through a robust antioxidant system, with glutathione (GSH) serving as a cornerstone molecule in ROS scavenging, cell cycle regulation, and anti-apoptotic signaling (24, 25). In both human ICP placentas and animal models of IUGR, glutathione peroxidase (GPx) activity is markedly diminished, indicating compromised antioxidant capacity (26). Our study corroborates these findings: placentas from cholestatic mice displayed significantly reduced total antioxidant capacity (T-AOC) and elevated malondialdehyde (MDA)-a hallmark of lipid peroxidation-providing direct evidence of oxidative damage.

This redox imbalance appears to stem, at least in part, from suppression of the Nrf2/Keap1 pathway, a master regulator of cellular antioxidant responses (27). Under oxidative stress, Nrf2 dissociates from its cytosolic inhibitor Keap1, translocates to the nucleus, and activates transcription of genes encoding GSH-synthesizing enzymes (e.g., GCLC, GCLM) and detoxifying proteins such as glutathione S-transferases (GSTs) and glutathione peroxidases (GPXs) (28–30). Notably, Nrf2 deficiency in mice results in impaired placental labyrinth development and reduced fetal growth (31, 32). In our model, RNA-seq revealed significant downregulation of multiple Nrf2-dependent antioxidant genes-including *Gstt1, Gsta4, Gstk1, Mgst1, Ggt1*, and *Gpx8*-suggesting that cholestasis disrupts this critical defense axis. We propose that bile acid accumulation during ICP suppresses Nrf2 signaling, thereby weakening placental antioxidant defenses and promoting oxidative injury that culminates in IUGR.

Beyond hepatic and placental effects, our data highlight a pivotal role for the maternal gut microbiota in the pathophysiology of gestational cholestasis (33). The microorganisms contribute to host health through various mechanisms, including vitamin synthesis, nutrient metabolism, and the secretion of small molecules involved in immune regulation, angiogenesis, and neuronal function (34, 35). The gut microbiome-a dynamic ecosystem essential for nutrient metabolism, immune education, and metabolite production-has emerged as a key modulator of pregnancy outcomes via the “gut–target organ axis” (36, 37). Dysbiosis during pregnancy has been linked to IUGR through mechanisms involving systemic inflammation and placental damage (38). In our DDC model, we observed profound microbial shifts: an increase in Escherichia-Shigella-an opportunistic pathogen associated with colitis and sepsis (39, 40)-and a depletion of beneficial taxa such as *Ruminococcus_torques_group* (negatively correlated with ICP risk (41)) and *Dubosiella* (known for anti-inflammatory properties (42)).

Critically, these microbial alterations coincided with reduced circulating levels of antioxidant metabolites, particularly valeric acid (a short-chain fatty acid, SCFA) and erythritol (43). SCFAs, produced by bacterial fermentation of dietary fiber, exert anti-inflammatory and antioxidant effects and have been shown to support early embryonic development (44, 45). Erythritol, meanwhile, exhibits direct ROS-scavenging activity and, as our molecular docking suggests, may activate the Nrf2 pathway by competitively binding Keap1 (46). The strong positive correlations between valeric acid/erythritol and commensal genera (*g__norank_f__Desulfovibrionaceae, g__unclassified_f__Lachnospiraceae*), coupled with their negative association with Akkermansia (which was elevated in DDC mice), further support a functional microbiota–metabolite–placenta axis.

## 5 Conclusion

In conclusion, this study delineates a novel mechanistic framework for ICP-associated IUGR: maternal cholestasis induces gut dysbiosis, which reduces the production of microbially derived antioxidant metabolites (e.g., valeric acid, erythritol). Concurrently, cholestasis suppresses placental Nrf2-driven antioxidant gene expression, leading to oxidative damage, placental insufficiency, and impaired fetal growth. Together, these findings position the “gut microbiota–metabolism–placenta” axis as a critical pathway in ICP pathogenesis and suggest that targeting microbial or redox homeostasis may offer therapeutic avenues to mitigate adverse pregnancy outcomes.

## Data Availability

The original contributions presented in the study are included in the article/supplementary material, further inquiries can be directed to the corresponding authors.
