# Supplementary material for: Mechanisms of fetal growth restriction in gestational cholestasis: role of gut microbiota and placental redox
Source: Front Vet Sci. 2026 Mar 20;13:1815498. doi: 10.3389/fvets.2026.1815498 (PMC13046547; doi:10.3389/fvets.2026.1815498)
Supplement: Supplementary file 1 [file Table_1.docx]

Supplementary Material

**Supplementary Table S1**

The main primers of q-PCR

| Gene | Sequences (Forward) | Sequences (Reverse) |
| --- | --- | --- |
| Mouse-*Mgst1*  (Gene ID: 56615) | CTCAGGCAGCTCATGGACAAT | GTTATCCTCTGGAATGCGGTC |
| Mouse-*Gsta4*  (Gene ID: 14860) | TTGAGACAAGGGAACAGTATGAGA | TGGAGCCACGGCAATCA |
| Mouse-*Gstt1*  (Gene ID: 14871) | CAGGCTCGTGCTCGTGTAGA | TGCCAGTGTTTCAGGAGGTATT |
| Mouse-*Ggt1*  (Gene ID: 14598) | TCTCCGAAGAGCGTAGCGA | GGGTGGGTGGTTTCATCAGT |
| Mouse-*Gpx8*  (Gene ID: 69590) | TTTCGCTGCCTACCCATTAA | TCCACCTTGGCTCCTTCTTG |
| Mouse-*Ggtk1*  (Gene ID: 69590) | ACTCCTGGCTGGGCTTTG | TTGGTTTCCGCTGTCTTTCAT |
| Mouse-*Actb*  (Gene ID: 11461) | GGCTGTATTCCCCTCCATCG | CCAGTTGGTAACAATGCCATGT |
